# Refractory reverse amblyopia with atropine penalization

**DOI:** 10.4103/0974-620X.71897

**Published:** 2010

**Authors:** Preeti Ajit Patil, S. Meenakshi, T. S. Surendran

**Affiliations:** Department of Pediatric Ophthalmology and Strabismus, Medical Research Foundation, Sankara Nethralaya, Chennai 600 006, India

**Keywords:** Atropine, penalization, reverse amblyopia

## Abstract

Pharmacological penalization with atropine has been shown to be equally effective as conventional occlusion therapy in the treatment of amblyopia in children. Reverse amblyopia of the sound eye with atropine penalization has been reported before, but is more common in cases where the effect is augmented with optical penalization and is mostly reversible. We report a case of reverse amblyopia with atropine penalization, in a 4-year-old girl, which was refractory to treatment. This report highlights the need for strict monitoring of the vision in the sound eye and regular follow-up in children undergoing amblyopia treatment.

## Introduction

Amblyopia is one of the commonest causes of visual impairment in children and is said to afflict 1–4% of children.[1] Occlusion therapy with patching of the sound eye has been the mainstay of amblyopia treatment. However, after the Amblyopia Treatment Study demonstrated that atropine penalization is of comparable effectiveness to patching for children with moderate amblyopia,[2,3] penalization has become an accepted modality of treatment of amblyopia.

Pharmacological penalization with atropine results in cycloplegia, prevents accommodation, and blurs the sound eye at near fixation. The effect may be augmented if the sound eye is hyperopic. Reverse amblyopia of the sound eye has been reported rarely and in most cases has been largely reversible.[1] However, this need not always be true and strict monitoring of the visual acuity in the better eye as well as compliance with regular follow-up schedules is a must.

We report a case of reverse amblyopia due to atropine penalization, which remained refractory to treatment.

## Case Report

A 4-year-old girl presented with occasional inward deviation of eyes, noticed since 3 years of age. On examination, her visual acuity was found to be 6/18 in the right eye (OD) and 6/24 in the left eye (OS), with unmaintained fixation for both distance and near OS. She also had a left esotropia of 20 PD. Her cycloplegic refraction was +1.25 DS OD and +2.50 DS OS. She was given the full cycloplegic correction and was advised part time occlusion OD, for 2 hours per day. At the next visit, 8 months later, it was noted that the visual acuity OD was 6/6 but OS was 6/24. The child was not compliant with the occlusion therapy. Atropine therapy was initiated, with the use of atropine eye ointment once a day OD. The patient was advised to continue using her glasses.

At the next follow-up visit, 3 months later, parents gave history of noticing inward deviation OD. On examination, the best-corrected visual acuity was 6/36 OD, and 6/9 OS, with unmaintained fixation in OD. Atropine therapy was discontinued, cycloplegic refraction was rechecked, and visual acuity retested. Her OD vision remained poor at subsequent visits and consequently, atropine therapy was started OS. She did not show a satisfactory response to penalization and was started on occlusion therapy with patching OS for 6 hours a day. Occlusion therapy was continued for over a year, but vision did not improve beyond 6/18 OD, whereas vision was maintained at 6/6 OS.

She underwent surgical correction of the esotropia (55 PD) with a medial rectus recession and lateral rectus resection in OD and a medial rectus recession OS. The occlusion therapy was continued after surgery, and she had a small residual esotropia of four PD for distance and near with a final visual acuity of 6/18 OD and 6/6 OS, at the final follow-up.

## Discussion

The Amblyopia Treatment Study demonstrated that atropine penalization is of comparable effectiveness to patching for children with moderate amblyopia.[2,3] However, the fact must be borne in mind that atropine penalization like conventional occlusion, comes with its own risks and clinically significant reverse amblyopia can occur, but is typically of very low incidence and transient, if treatment is discontinued, or reversible.[1]

In the Amblyopia Treatment Study, 47 of 204 patients, treated with atropine lost one or more lines of vision in the sound eye by their 6-month follow-up examination. This was largely attributed to the cycloplegic effect of the atropine and incorrect spectacle correction. Follow-up examination was performed in 45 of these 47 patients. Visual acuity had returned to baseline in 40, while five patients had a residual decrease of one line. Only one patient required active treatment for decreased visual acuity.[4]

In another trial, which compared patching with atropine penalization for the treatment of moderate amblyopia in 88 children aged 7–12 years, no participant was diagnosed with reverse amblyopia after 17 weeks of follow-up.[5]

A similar study which compared weekend atropine use augmented by a plano lens for the sound eye with weekend atropine use alone for moderate amblyopia in children aged 3–7 years, also found no cases of persistent amblyopia in the 180 children studied, after 18 weeks of follow-up.[6]

Reverse amblyopia with atropine has been reported previously,[7,8] but is more likely if atropine penalization is combined with optical penalization.

Morrison *et al*. reported two cases of reverse amblyopia as a result of noncompliance during atropine therapy combined with optical penalization.[9] Both cases required active treatment. Vision returned to normal in one patient, while the second patient was lost to follow up.

Hainline *et al*. studied 133 patients with amblyopia who were treated with only atropine penalization or occlusion or a combination of penalization and occlusion. Of 133 patients, 8 developed reverse amblyopia. All of them were successfully treated, with six patients requiring only discontinuation of atropine and two requiring patching to correct the reverse amblyopia.[10]

In our case, reverse amblyopia occurred in 3 months of starting atropine penalization, even when not augmented by optical penalization. In addition, despite aggressive treatment, visual acuity did not return to baseline. It is unlikely that decreased visual acuity OD was due to incorrect spectacles or inadequate cycloplegia as the cycloplegic refraction was repeated and the visual acuity was rechecked with full hyperopic correction, as per the ATS protocol in cases of suspected reverse amblyopia.

Various other side effects have been noted with atropine penalization especially facial flushing, fever, irritation, and light sensitivity.[1] None of these were seen in our patient, but the treating ophthalmologist must be aware of these potential side effects.

This case report highlights the need for regular follow-up and careful monitoring of a fixation switch or a drop in vision in the good eye while the child is on penalization therapy with atropine. Atropine penalization is a very effective modality of amblyopia treatment, but reverse amblyopia may set in, in a relatively short duration and may be irreversible.
